# Methanol Extract from *Anogeissus leiocarpus* (DC) Guill. et Perr. (Combretaceae) Stem Bark Quenches the Quorum Sensing of *Pseudomonas aeruginosa* PAO1

**DOI:** 10.3390/medicines3040026

**Published:** 2016-10-06

**Authors:** Vincent Ouedraogo, Martin Kiendrebeogo

**Affiliations:** Laboratoire de Biochimie & Chimie Appliquées, Université Ouaga 1 Pr. Joseph KI-ZERBO, Ouagadougou, 03 BP 7021, Burkina Faso; vicenteoued@gmail.com

**Keywords:** *Anogeissus leiocarpus*, *Chromobacterium violaceum* CV026, *Pseudomonas aeruginosa* PAO1, pyocyanin, quorum sensing

## Abstract

**Background:** Due to its extensive arsenal of virulence factors and inherent resistance to antibiotics, *Pseudomonas aeruginosa* is a threat particularly in immunocompromised patients. Considering the central role of quorum sensing in the production of virulence factors, inhibition of bacterial communication mechanism constitute an opportunity to attenuate pathogenicity of bacteria resistant to available antibiotics. Our study aimed to assess the anti-quorum sensing activity of *Anogeissus leiocarpus*, traditionally used in Burkina Faso, for the treatment of infected burn wounds. **Methods:** Investigations were carried out on methanol extract from *A. leiocarpus* stem bark. The reporter strains *Chromobacterium violaceum* CV026 and *P. aeruginosa* PAO1 derivatives were used to evidence any interference with the bacterial quorum sensing and expression of related genes. *P. aeruginosa* PAO1 was used to measure the impact on pyocyanin production. **Results:** At a sub-inhibitory concentration (100 µg/mL), *A. leiocarpus* methanol extract quenched the quorum sensing mechanism of *P. aeruginosa* PAO1 by down-streaming the *rhlR* gene, with a subsequent reduction of pyocyanin production. Moreover, the antioxidant polyphenols evidenced are able to reduce the oxidative stress induced by pyocyanin. **Conclusion:** The antioxidant and anti-quorum sensing activities of *A. leiocarpus* stem bark could justify its traditional use in the treatment of infected burn wounds.

## 1. Introduction

*Pseudomonas aeruginosa* infections are common following burn injuries [1] and often present as wound infections [2]. Due to its extensive arsenal of virulence factors and inherent resistance to several antibiotics, approximately 80% of burn patients infected with *P. aeruginosa* die of septicaemia [3]. Production of virulence factors by *P. aeruginosa* is under the control of a cell-to-cell communication system termed quorum sensing (QS), which is a mechanism used by many bacteria to detect their critical cell numbers through the release and perception of small diffusible signal molecules called auto inducers, in order to coordinate a common behavior [4,5,6].

In *P. aeruginosa*, two QS systems (lasI/lasR and rhlI/RhlR) drive the production (by the synthetases LasI and RhlI) and the perception (by the transcription factors LasR and RhlR) of the auto inducers acyl homoserine lactones (AHL)—*N*-(3-oxododecanoyl)-l-homoserine lactone (3-oxo-C12-HSL) and *N*-butanoyl-l-homoserine lactone (C4-HSL), respectively [4,5,7,8]. A third QS system, based on quinolone signals, links and interacts in an intricate way with the lasI/lasR and rhlI/RhlR quorum-sensing systems [9,10,11,12].

Following injuries, inflammation process (representing a non-specific immune response to chemical or biological aggression) takes place in order to pathogen elimination and tissue injury reparation [13,14,15].

Inflammatory reactions start with the release of inflammatory mediators (e.g., cytokines, endotoxins, prostaglandins, leukotrienes, and histamine) as well as reactive oxygen species (ROS) by injured cells.

In non-pathogenic conditions, cells are normally able to defend themselves against ROS damage through the use of endogen enzymatic and non-enzymatic antioxidants agents. Since *P. aeruginosa* infection during a burn wound dramatically boosts the level of ROS, antioxidant defense systems of cells are overpassed, resulting in oxidative stress with significant damage to cell structures [16,17].

In the ongoing struggle against bacterial infection, antibiotics are commonly used to kill pathogenic bacteria. However, bacteria increasingly exhibit resistance against available antimicrobial drugs [18,19]. To cope with these limitations, the alternative approach consisting in attenuating the expression of bacterial virulence factor without affecting their viability by using anti-QS agents has become a rational preventive strategy [20,21].

In Burkina Faso, more than 80% of the population relies on traditional practices and medicinal plants to treat various diseases [22]. Some medicinal plants have been reported to present anti-inflammatory activity and wound healing effects without any antibacterial activity [22,23], which suggests a non-antimicrobial modulation of virulence factors.

In this study, *Anogeissus leiocarpus* (DC) Guill. & Perr. (Combretaceae), which is traditionally involved in Burkina Faso for the treatment of infected burn wounds [22], was investigated for its ability to reduce the production of pyocyanin, one of the QS-controlled virulence factors produced by *P. aeruginosa*, and to interfere with the bacterial QS system. Total polyphenol and flavonoid content as well as the antioxidant potentiality of this medicinal plant were also assessed.

## 2. Materials and Methods

### 2.1. Bacterial Strains, Plasmids, and Culture Conditions

*Chromobacterium violaceum* CV026, *Pseudomonas aeruginosa* PAO1, and its derivatives (Table S1) harboring plasmids pPCS1001 (P*_lasR_-lacZ* transcriptional fusion), pβ03 (P*_lasI_-lacZ* transcriptional fusion), pPCS1002 (P*_rhlR_-lacZ* transcriptional fusion), pLPR1 (P*_rhlI_-lacZ* transcriptional fusion), pTB4124 (P*_acAe_-lacZ* transcriptional fusion) and pβ02 (P*_rhlA_-lacZ* transcriptional fusion) were provided from the Laboratoire de Biotechnologie Vegetale (Université Libre de Bruxelles, Gosselies, Belgium). *P. aeruginosa* PAO1 (37 °C, agitation 175 rpm) and *C. violaceum* CV026 (30 °C, agitation 175 rpm) were grown in LB broth. *P. aeruginosa* PAO1 derivatives strains were grown (37 °C, agitation 175 rpm) in LB-MOPS broth (50 mM, pH 7) supplemented with carbenicillin (300 µg/mL).

### 2.2. Plant Material Collection and Extraction

Stem bark of *Anogeissus leiocarpus* (DC) Guill. et Perr. (Combretaceae) was collected in August 2014 at Gampela (25 km, east of Ouagadougou, Burkina Faso). Botanical identity was assessed by Dr. Amade Ouedraogo from the Laboratoire de Biologie et Ecologie Vegetale (Université Ouaga 1 Pr. Joseph Ki-Zerbo, Ouagadougou, Burkina Faso) where a voucher specimen (ID: 16883) was deposited. Plant material was dried at room temperature, ground into fine powder, and stored in an airtight bag until use.

Powdered plant material (100 g) was defatted with petroleum ether (500 mL) in a soxhlet extractor (Wheaton industries Inc., Millvile, NJ, USA) and soaked (24 h, 25 °C, continuous stirring) in methanol. Extract was filtrated, concentrated in a vacuum evaporator (Büchi Labortechnik AG, Postfach, Flawil, Switzerland) and dried to obtain 14.4 g of plant extract. Plant extract (100 mg/mL) was dissolve either in methanol for phytochemical purpose or in dimethyl sulfoxide (DMSO) for testing on bacterial strains. Test samples were stored at 4 °C until use.

### 2.3. Total Polyphenol and Flavonoid Content Determination

Total polyphenol was determined according to the colorimetric method of Folin–Ciocalteu [24]. Plant extract (25 µL, 100 µg/mL in methanol) was mixed with Folin–Ciocalteu Reagent (125 µL, 0.2 N) and, 5 min later, with sodium bicarbonate (100 μL, 75 g/L). After incubation (1 h, room temperature), absorbance was measured at 760 nm against a methanol blank. Gallic acid (0–100 mg/L) was used to generate a standard calibration curve (*Y* = 0.005*X* + 0.00968; *R*^2^ = 0.99), and total polyphenol content was expressed as mg gallic acid equivalent to 100 mg of plant extract (mg GAE/100 mg).

Total flavonoid was estimated according to the Dowd method [24]. Plant extract (75 µL, 100 µg/mL in methanol) was mixed with aluminium trichloride (75 µL, 2% in methanol). Absorbance was subsequently read at 415 nm after incubation (10 min, room temperature) against a methanol blank. Quercetin (0–100 mg/L) was used to plot a standard calibration curve (*Y* = 0.02891*X* + 0.0036; *R*^2^ = 0.99), and total flavonoid content was expressed as mg of quercetin equivalent to 100 mg of plant extract (mg QE/100 mg).

### 2.4. Antioxidant Assays

Antioxidant activity was measured through 2,2-diphenyl-1-picrylhydrazyl (DPPH) and ferric reducing antioxidant power (FRAP) assays as previously described [24].

For the DPPH assay, freshly prepared DPPH solution (200 µL, 0.02 mg/mL in methanol) was mixed with plant extract (100 µL, 100 to 0.39 µg/mL in methanol). The mixture was subsequently shacked and incubated (15 min in darkness, room temperature), and absorbance was measured at 517 nm against a methanol blank. DPPH radical scavenging activities were plotted against sample concentrations, and the result was expressed as a sample concentration scavenging 50% of DPPH radicals (IC50). Quercetin was used as positive controls.

For FRAP testing, plant extract (100 µL, 100 µg/mL in methanol) was mixed with a phosphate buffer (250 µL, 0.2 M, pH 6.6) and a potassium hexacyanoferrate solution (250 µL, 1% in water). After incubation (30 min, 50 °C), trichloroacetic acid (250, 10% in water) was added, and the mixture centrifuged (2000× *g* for 10 min). The supernatant (125 µL) was mixed with water (125 µL) and a fresh FeCl_3_ solution (25 µL, 0.1% in water) to read absorbance at 700 nm. Ascorbic acid was used to plot a calibration curve (*R*^2^ = 0.99). Reducing power was expressed as µM ascorbic acid equivalent per gram of plant extract (µM AAE/g). Quercetin was used as positive controls.

### 2.5. Determination of MIC and MBC

MIC (minimum inhibitory concentration) values on *P. aeruginosa* PAO1 and *C. violaceum* CV026 were determined according to the micro dilution method, using *p*-iodonitrotetrazolium (INT) as an indicator of bacterial growth [25]. In brief, an overnight bacterial culture was diluted with LB broth to obtain a starting inoculum (109 CFU/mL). Inoculum (180 μL) was added to serial dilutions of test extract (20 μL; 50 to 0.39 mg/mL in DMSO 10%) to obtain a final concentration range of 5 mg/mL to 0.039 mg/mL. Mixtures were incubated for 18 h (37 °C, 175 rpm agitation). After 18 h of incubation, 50 μL of INT (0.2 mg/mL) was added to each well, and the microplate was further incubated (37 °C, 30 min). Bacterial growth was indicated by a red color within the microplate wells (Greiner Bio-One GmbH, Frieckenhausen, Germany).

To determine the MBC, aliquots of 20 μL from all dilutions not showing any bacterial growth were spread onto LB agar plates and incubated (37 °C, 24 h). The minimum concentration for which there is no visible growth on agar plate was recorded as MBC [26].

### 2.6. Quantitative Analysis of Pyocyanin Production in P. aeruginosa PAO1

Inhibition of pyocyanin production was assessed according to previously described procedures [27]. *P. aeruginosa* PAO1 was grown (18 h in LB broth, 37 °C, 175 rpm agitation), and cells were washed twice in fresh LB medium. In 18 culture tubes, appropriately diluted PAO1 cell suspension (250 µL) was added to the LB medium (4.7 mL, starting OD600nm ranged between 0.02 and 0.03) and supplemented with plant extract (50 µL, 10 mg/mL in DMSO) or DMSO. At periodic time intervals (3 h), tubes (*n* = 3) were sampled to assess bacterial growth (ufc/mL) and pyocyanin content.

From each tube, 100 µL of bacterial culture were removed and diluted in LB broth to be plated onto LB agar and incubated (24 h, 37 °C) for colony counting, while 200 µL of bacterial culture was used to determine optical density at 600 nm. Remaining bacterial culture was centrifuged (7000 rpm, 10 min, 24 °C) to obtain culture supernatant. Pyocyanin was extracted from the supernatant (4 mL) with chloroform (2 mL) and re-extracted from chloroform with 0.2 M HCl (1 mL). Optical density reading at 380 nm allows pyocyanin determination.

### 2.7. Quantitative Analysis of Violacein Production in C. violaceum CV026

Inhibition of violacein production in *C. violaceum* CV026 was tested according to [27]. Violacein production was induced in *C. violaceum* CV026 by adding exogenous *N*-hexanoyl-l-homoserine lactone (HHL; Sigma-Aldrich Chemie GmbH, Darmstadt, Germany).

An appropriately diluted overnight culture of *C. violaceum* CV026 (200 µL) was incubated (30 °C, 48 h, 175 rpm agitation) in 18 culture tubes containing LB broth (4.7 mL) supplemented with HHL (50 µL, 10 mM in DMSO) and plant extract (50 µL, 10 mg/mL in DMSO) or DMSO. At periodic time intervals (6 h), tubes (*n* = 3) were sampled to assess bacterial growth (ufc/mL and OD600 nm) and pyocyanin content, while violacein was quantified after 48 h growth.

From each tube, bacterial culture (1 mL) was centrifuged (7000 rpm, 10 min) and DMSO (1 mL) was added to the pellet. The solution was vortexed to dissolve violacein, and cell debris was discarded by centrifugation (7000 rpm, 10 min). Violacein content in supernatant was measured by the absorbance at 585 nm.

### 2.8. β-Galactosidase Assay

β-Galactosidase measurements were performed as previously described [27]. After growth in liquid LB-MOPS-Carbenicillin (37 °C with 175 rpm agitation) for 18 h, PAO1 reporter strains harboring plasmids (Table S1) were washed twice in fresh LB medium and resuspended in fresh liquid LB-MOPS-Carbenicillin. PAO1 reporter strains inoculums (50 µL) were incubated (37 °C with 175 rpm agitation) for 8 or 18 h in 1 mL of LB-MOPS-Carbenicillin (initial OD600 nm of culture comprised between 0.020 and 0.025) supplemented with 10 µL of plant extracts (100 µg/mL final concentration) or 10 µL of DMSO. After incubation, bacterial density was assessed by spectrophotometry (OD600 nm), and the sample used for cell growth assessment was used to perform the β-galactosidase assay with *o*-nitrophenyl-β-d-galactopyranoside as described elsewhere [28].

### 2.9. Statistical Analysis

All experiments were performed in triplicate (independent assays), and data were expressed as mean ± SD. Data analysis was performed via analysis of variance (one-way ANOVA or two-way ANOVA) followed by a Tukey or Bonferonni test, using GraphPad Prism software (version 5.00 for window, GraphPad Software, San Diego, CA, USA) *p* value ≤ 0.05 was considered significant.

## 3. Results

### 3.1. Antioxidant Activity, Total Polyphenol, and Flavovoid Content

Total polyphenol and total flavonoid were quantified from methanol extract of *A. leiocarpus* stem bark together with its antioxidant capacity through radicals DPPH scavenging activity and ferric reducing power, and each antioxidant assay involved a different antioxidant mechanism. The amount of total polyphenol was particularly high (82.62 ± 3.16 mg GAE/100 mg) in addition to a low content of total flavonoid (15.14 ± 0. 39 mg QE/100 mg). As shown, an interesting antioxidant potential was found. *A. leiocarpus* exhibits the same DPPH radical scavenging activity (1.82 ± 0.07 µg/mL) as quercetin (1.40 ± 0.15 µg/mL), while its ferric-reducing power (4.29 ± 0.19 µM AAE/g) was only two-fold lower than that of our antioxidant reference (7.66 ± 0.39 µM AAE/g).

### 3.2. Inhibition of Pyocianin Production

**Figure 1:**
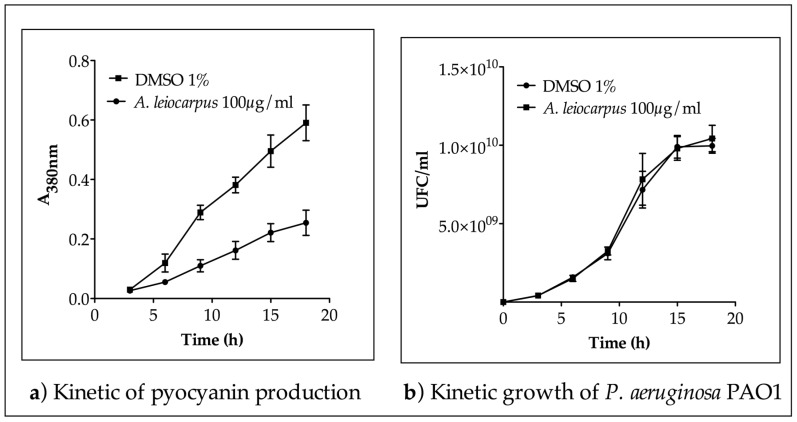
Methanol extract from *A. leiocarpus* reduce pyocyanin production in *P. aeruginosa* PAO1. (**a**) *A. leiocarpus* extract (100 µg/mL) significantly reduced (*p* > 0.05) pyocyanin production within 18 h compared to DMSO used as control; (**b**) *A. leiocarpus* extract (100 µg/mL) did not exhibit a significant effect on *P. aeruginosa* PAO1 kinetic growth within 18 h. Dimethyl sulfoxide (DMSO) was used as negative control. *P. aeruginosa* PAO1 was grown (37 °C, 175 rpm agitation) in the LB broth. Mean values ± SD of triplicate independent experiments are shown.

The MIC (1.25 mg/mL) and MBC (>5.00 mg/mL) values evaluated allow for the selection of a sub-inhibitory concentration for the bioassay on *P. aeruginosa* PAO1. Methanol extract from *A. leiocarpus* stem bark (100 µg/mL) was incubated for 18 h in *P. aeruginosa* PAO1 culture to access its capacity to interfere with the production of pyocyanin, a QS-dependent extracellular virulence factor of the bacteria. As shown, methanol extract from *A. leiocarpus* (100 µg/mL final concentration) significantly impact (*p* < 0.05) in a kinetic way the production of pyocyanin (Figure 1a) without any effect on bacterial kinetic growth (Figure 1b). Hence, the reduction of pyocyanin production recorded within 18 h was not the consequence of any bactericidal or bacteriostatic effect but probably the effect of some interference with the QS mechanism of *P. aeruginosa* PAO1, controlling the production of pyocyanin.

### 3.3. Anti-Quorum Sensing Activity

**Figure 2:**
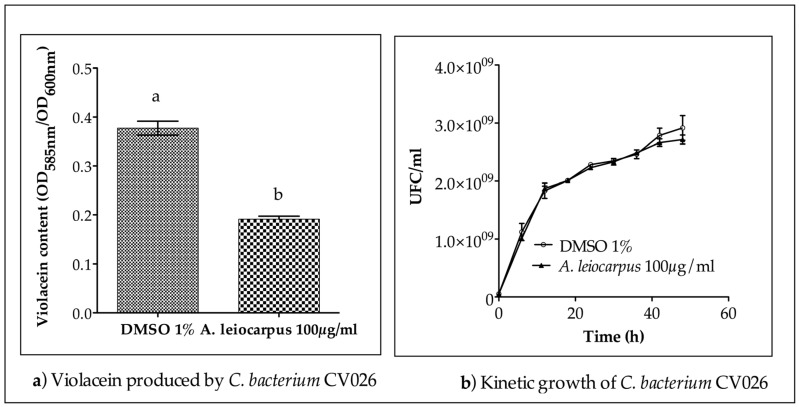
Anti-quorum sensing (QS) activity of methanol extract from *A. leiocarpus*. (**a**) *A. leiocarpus* extract (100 µg/mL) significantly reduces (*p* > 0.05) violacein production by *C. violaceum* CV026 within 48 h (**b**) *A. leiocarpus* extract (100 µg/mL) did not exhibit a significant effect of on *C. violaceum* CV026 kinetic growth within 48 h. Dimethyl sulfoxide (DMSO) was used as a negative control. *C. violaceum* CV026 was grown (30 °C, 175 rpm agitation) in LB broth supplemented with HHL (10 µM in DMSO). Mean values of triplicate independent experiments and SD are shown. Data with different letters in superscript are significantly different (*p* > 0.05).

To assess the ability of the methanol extract from *A leiocarpus* stem bark to interfere with the quorum sensing mechanism, the reporter strain *C. violaceum* CV026, deficient in the homoserine-lactone synthase gene *cviI*, was used. This strain is unable to produce quorum sensing auto inducers (homoserine-lactones) by itself, nor therefore the QS-related violacein, without an external supply of homoserine-lactone. The MIC (0.62 mg/mL) and MBC (2.50 mg/mL) values evaluated allow for the selection of a sub-inhibitory concentration for the bioassay on *C. violaceum* CV026. Methanol extract from *A. leiocarpus* (100 µg/mL final concentration) reduced violacein production by up to 50% (Figure 2a) without any effect on bacterial kinetic growth (Figure 2b). Therefore, the reduction of violacein production observed was not due to any bactericidal or bacteriostatic effect, but to a quenching of the QS system of the bacteria. When extract was added to *C. violaceum* CV026 growth medium without a HHL supply, violacein was not produced, indicating that *A. leiocarpus* extract do not contain mimic HHL compound (data not shown).

**Figure 3:**
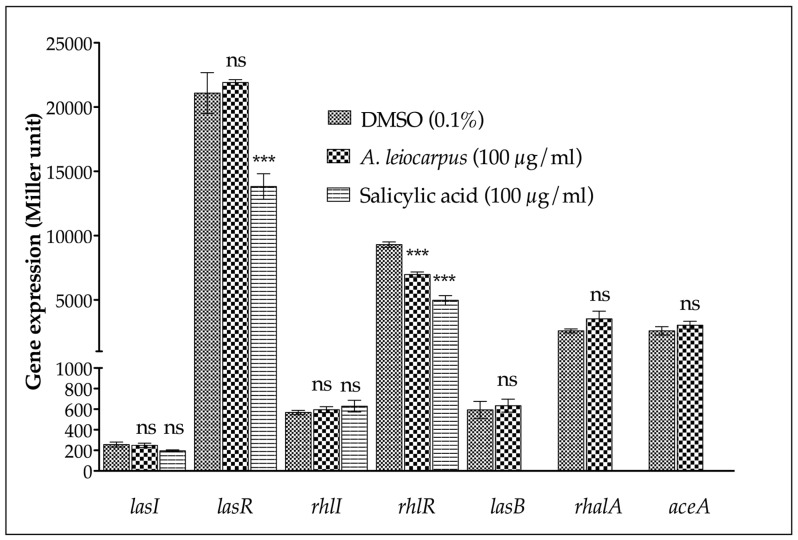
Effect of *A. leiocarpus* extract on the transcription level of *lasRI* and *rhlRI* QS genes. Data are expressed as mean ± SD of 3 independent essays. Dimethyl sulfoxide (DMSO) was used as negative control. Salicylic acid was used as positive control. *** Significantly different compared with DMSO treatment (*p* < 0.05). ns: not significantly different compared to DMSO treatment. Gene expression was measured as the β-galactosidase activity of the *lacZ* gene fusions and expressed in Miller units. Expression of the *aceA* gene is used as a quorum-sensing independent control. *P. aeruginosa* PAO1 strains were grown.

In order to evidence any interference with the expression of quorum sensing (QS) genes of *P. aeruginosa* PAO1, we focused on the transcriptional level after 18 h of incubation. Therefore, the expression of the HHL synthetases genes (*lasI* and *rhlI*), the QS regulator genes (*lasR* and *rhlR*), and the virulence factor QS-controlled genes (*lasB* and *rhlA*) were evaluated. As shown (Figure 3), methanol extract of *A. leiocarpus* stem bark did not significantly affect (*p* < 0.05) the transcription level of the quorum sensing *lasI*, *lasR*, and *rhlI* genes, while expression of *rhlR* was significantly (*p* < 0.05) downstreamed.

To determine whether the drop in β-galactosidase activity recorded was not due to an effect on the transcription/translation mechanisms, nor to an inhibition of the enzyme (β-galactosidase), a *P. aeruginosa* PAO1 strain harboring the aceA-lacZ fusion (*aceA* gene encoding for isocitrate lyase) was tested. As shown, *A. leiocarpus* extract had no effect on the transcription of the *aceA* gene, demonstrating that it affects the expression of QS-related genes without interfering with the entire transcription machinery of *P. aeruginosa* PAO1. Hence, *A. leiocarpus* methanol extract clearly exhibited anti-QS activity on *P. aeruginosa* PAO1.

## 4. Discussion

Our in vitro investigations show that methanol extract from *A. leiocarpus* stem bark exhibits promising antioxidant capacity along with anti-QS activity with a subsequent reduction of pyocyanin production. Since *phz*, the biosynthesis genes of pyocyanin, is under the control of *las* and *rhl* QS systems [29,30], the observed decrease in pyocyanin production is related to the downstreaming of the QS regulator gene *rhlR*.

*P. aeruginosa* PAO1 QS systems *las* and *rhl* seems to be important for a successful infection in burn wounds, as the virulence of PAO1 mutants defective in either *lasI*, *lasR*, or *rhlI* is reduced [31]. Consistent with this, the inability of QS-deficient strains to induce a successful infection was proposed to be linked with a decreased production of virulence factors such as pyocyanin among other virulence factors, and the most significant virulence reduction was detected with the mutant defective in both *lasI* and *rhlI* [31]. Hence, the anti-QS demonstrated might explain the traditional usage of *A. leiocarpus* stem bark to treat infected burn wounds.

Within the Combretaceae family, to which belongs *A. leiocarpus*, anti-QS medicinal plants have been previously reported. *Bucida buceras*, *Combretum albiflorum*, and *Conocarpus erectus* quench the QS mechanism of *P. aeruginosa* PAO1 with a subsequent inhibition of the bacteria virulence factors [27,32]. Polyphenols and flavonoids are also known for their anti-QS potentiality. The flavonoids catechin, isolated from *Combretum albiflorum* and naringenin have been reported to inhibit QS-regulated virulence factors expression in *P. aeruginosa* and thought to possibly interfere with the perception of the native AHL by LasR and RhlR [27,33]. Epigallocatechin gallate, tannic acid, and ellagic acid also demonstrated their anti-QS potential against *Pseudomonas putida* [34]. Vescalagin and castalagin, two ellagitannins isolated from *Conocarpus erectus*, decrease AHL production, QS gene expression and elastases production [35]. The anti-QS activity and related reduction of pyocyanin production observed within our study might be due to castalagin, since it has been previously isolated from the stem bark of *A. leiocarpus* [36]. Furthermore, 3,3′,4′-tri-*O*-methylflavellagic acid, 3,3′-di-*O*-methylellagic acid, and 3,4,3′-tri-*O*-methylflavellagic acid-4′-β-d-glucoside, three ellagic acid derivatives isolated from *A. leiocarpus* stem bark [37], might contribute to the anti-QS bioactivity we demonstrated.

Among several virulence factors produced by *P. aeruginosa*, pyocyanin is known to be involved in cell host degradation and the production of ROS [38,39]. Pyocyanin alters the redox cycle involved in cellular respiration and increases the oxidative stress on host cells [1]. The mechanism by which pyocyanin causes cell cycle arrest is related to its redox (oxidation–reduction) properties, and its ability to reduce molecular oxygen into reactive oxygen species induces low-level, yet persistent, oxidative stress [40,41]. Thus, pyocyanin is able to delay cicatrization and might also lead to chronic inflammation in wound and burn injuries infected by *P. aeruginosa*. Polyphenols and flavonoids responsible for the antioxidant activity of *A. leiocarpus* could therefore contribute to the reduction of the oxidative stress caused by pyocyanin and thus reduce inflammatory intensity independently of pyocyanin reduction, a benefit for wound healing.

Taken together, anti-QS and antioxidant activities of *A. leiocarpus* could contribute to an explanation of its wound-healing benefit. The antioxidant activities could represent a preventive action to minimize ROS due to inflammation but also counterbalance the high level production of ROS when burn injuries are infected by opportunistic pathogens. Additionally, the anti-QS activity also preserves a minimal ROS level as it reduces virulence factor production such as pyocyanin, and more importantly reduce the abilities of pathogens to degrade host tissue and to resist host immune responses that can maintain inflammation and delay the healing process.

## 5. Conclusions

Our study demonstrated the antioxidant and anti-QS activities of the methanol extract from *A. leiocarpus* stem bark. Based on bibliographic reports, catalagin and ellagic acid derivatives might be responsible for the anti-QS property demonstrated. These results contribute to the establishment of the traditional use of *A. leiocarpus* stem bark in the management of infected burn wounds on a rational basis. By reducing the production of pyocyanin and related oxidative stress within infected tissues in a QS manner, *A. leiocarpus* stem bark benefits to the healing process of septic injuries.

In future investigations, we will focus on the interference of the anti-QS molecules from *A. leiocarpus* either with the mechanisms of perception or production of homoserine lactones (lasI/lasR, rhlI/rhlR QS systems) or with the quinolone signal (the third QS system) within *P. aeruginosa*.

## Supplementary Materials

The following are available online at www.mdpi.com/2305-6320/3/4/26/s1. Table S1: List of plasmids used in this study.
