# Supplementary material for: Methanol Extract from Anogeissus leiocarpus (DC) Guill. et Perr. (Combretaceae) Stem Bark Quenches the Quorum Sensing of Pseudomonas aeruginosa PAO1
Source: Medicines (Basel). 2016 Oct 6;3(4):26. doi: 10.3390/medicines3040026 (PMC5456239; doi:10.3390/medicines3040026)
Supplement: Supplementary file 1 [file medicines-03-00026-s001.pdf]

Supplementary Materials: Methanol Extract from *Anogeissus leiocarpus* (DC) Guill. et Perr. (Combretaceae) Stem Bark Quench the Quorum Sensing of *Pseudomonas aeruginosa* PAO1

Vincent Ouedraogo and Martin Kiendrebeogo

Table S1. List of plasmids used in this study.

Plasmids	Description	References
pLP170	Broad-host-range *lacZ* transcriptional fusion vector that contains an RNase III splice sequence positioned between the multiple cloning site and *lacZ*; Cb^r	[1]
pPCS1001	pLP170-derivative containing P*lasR-lacZ* transcriptional fusion	[1]
pPCS1002	pLP170-derivative containing P*rhlR-lacZ* transcriptional fusion	[1]
pLPR1	pLP170-derivative containing P*rhlI-lacZ* transcriptional fusion	[2]
pQF50	Broad-host-range promoter-less *lacZ* transcriptional fusion vector; Cb^r	[3]
pβ03	pQF50-derivative containing P*lasI-lacZ* transcriptional fusion	[3]
pTB4124	pQF50 *lacZ* transcriptional fusion derivative containing P*aceA*	[4]

Cb^r, carbenicillin resistance.

Reference

1. Pesci, E.C.; Pearson, J.P.; Seed, P.C.; Iglewski, B.H. Regulation of *las* and *rhl* quorum sensing in *Pseudomonas aeruginosa*. *J. Bacteriol.* **1997**, *179*, 3127–3132.
2. Van Delden, C.; Pesci, E.C.; Pearson, J.P.; Iglewski, B.H. Starvation selection restores elastase and rhamnolipid production in a *Pseudomonas aeruginosa* quorum-sensing mutant. *Infect. Immun.* **1998**, *66*, 4499–4502.
3. Ishida, T.; Ikeda, T.; Takiguchi, N.; Kuroda, A.; Ohtake, H.; Kato, J. Inhibition of quorum sensing in *Pseudomonas aeruginosa* by *N*-acyl cyclopentylamides. *Appl. Environ. Microbiol.* **2007**, *73*, 3183–3188.
4. Kretzschmar, U., Khodaverdi, V.; Jeoung, J.H.; Gorisch, H. Function and transcriptional regulation of the isocitrate lyase in *Pseudomonas aeruginosa*. *Arch. Microbiol.* **2008**, *190*, 151–158.
